# Immune‐related adverse events in hepatitis treated with thiopurine‐based immunosuppressants: A case report

**DOI:** 10.1111/1759-7714.14859

**Published:** 2023-03-19

**Authors:** Keita Kawakado, Kazuhisa Nakashima, Hiroshi Tobita, Mamiko Nagase, Ken Yoshihara, Mika Horie, Megumi Hamaguchi, Tamio Okimoto, Yukari Tsubata, Takeshi Isobe

**Affiliations:** ^1^ Department of Internal Medicine, Division of Medical Oncology and Respiratory Medicine Shimane University Faculty of Medicine Izumo Japan; ^2^ Department of Gastroenterology and Hepatology Shimane University Faculty of Medicine Izumo Japan; ^3^ Department of Organ Pathology Shimane University Faculty of Medicine Izumo Japan; ^4^ Department of Respiratory Internal Medicine Oda Municipal Hospital Oda Japan

**Keywords:** azathioprine, hepatitis, immune‐related events, mercaptopurine

## Abstract

An 82‐year‐old man was treated with ipilimumab and nivolumab for malignant pleural mesothelioma. Although he was previously treated with prednisolone (1 mg/kg/day) for immune‐related adverse event (irAE) hepatitis by a previous doctor, he still had worsening liver function and was transferred to our hospital. Blood tests and imaging findings were negative for autoimmune and infectious hepatitis, and liver biopsy results were consistent with irAE hepatitis. Steroid pulse therapy improved liver function, but tapering to prednisolone (1 mg/kg/day) again worsened his liver function. Concomitant use of mycophenolate mofetil was initiated, but no improvement in liver function was observed, therefore azathioprine, a thiopurine immunosuppressant, was administered in combination with steroids. During the course of treatment, hepatic dysfunction due to azathioprine was suspected, and the concomitant use of mercaptopurine and prednisolone was started. Afterward, the liver function improved, and the prednisolone dose was gradually reduced to 10 mg/day. This is a rare case in which a thiopurine‐based immunosuppressant was effective against irAE hepatitis, therefore thiopurine‐based immunosuppressants may be effective against steroid‐refractory hepatitis.

## Graphical abstract

Coarse of treatment time from initiation of ipilimumab + nivolumab and changes in liver function, aspartate aminotransferase, alanine aminotransferase, and total bilirubin. Steroid pulse therapy means 3‐day administration of 1000 mg of methylprednisolone.

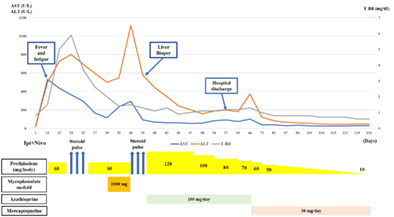

## INTRODUCTION

The efficacy of nivolumab, a fully human antiprogrammed death‐1 antibody, and ipilimumab, a fully human anticytotoxic T‐lymphocyte antigen‐4 antibody, has been reported for the treatment of unresectable malignant pleural mesothelioma. However, hepatitis, an immune‐related adverse event (irAE), occurred in approximately 6.0% of common terminology criteria for adverse events (CTCAE) of any grade and 4.7% of grades 3 and 4.^1^ Herein, we report a case in which only mycophenolate mofetil was not effective against steroid‐refractory irAE hepatitis, and thiopurine‐based immunosuppressants were co‐administered to improve the patient's condition.

## CASE REPORT

**FIGURE 1:**
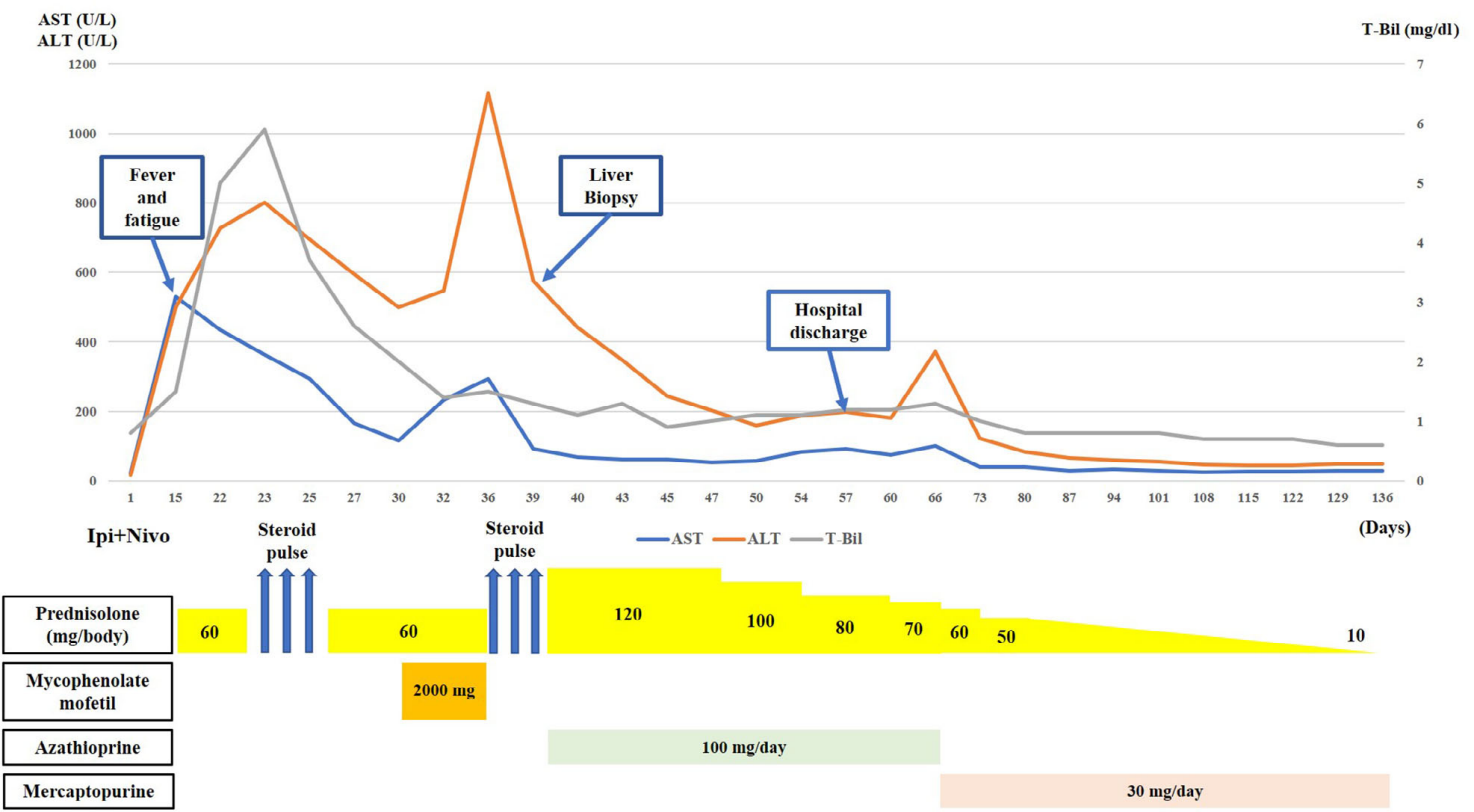
Coarse of treatment time from initiation of ipilimumab (Ipi) + nivolumab (Nivo) and changes in liver function, aspartate aminotransferase (AST), alanine aminotransferase (ALT), and total bilirubin (T‐Bil). Steroid pulse therapy means 3‐day administration of 1000 mg of methylprednisolone.

**FIGURE 2:**
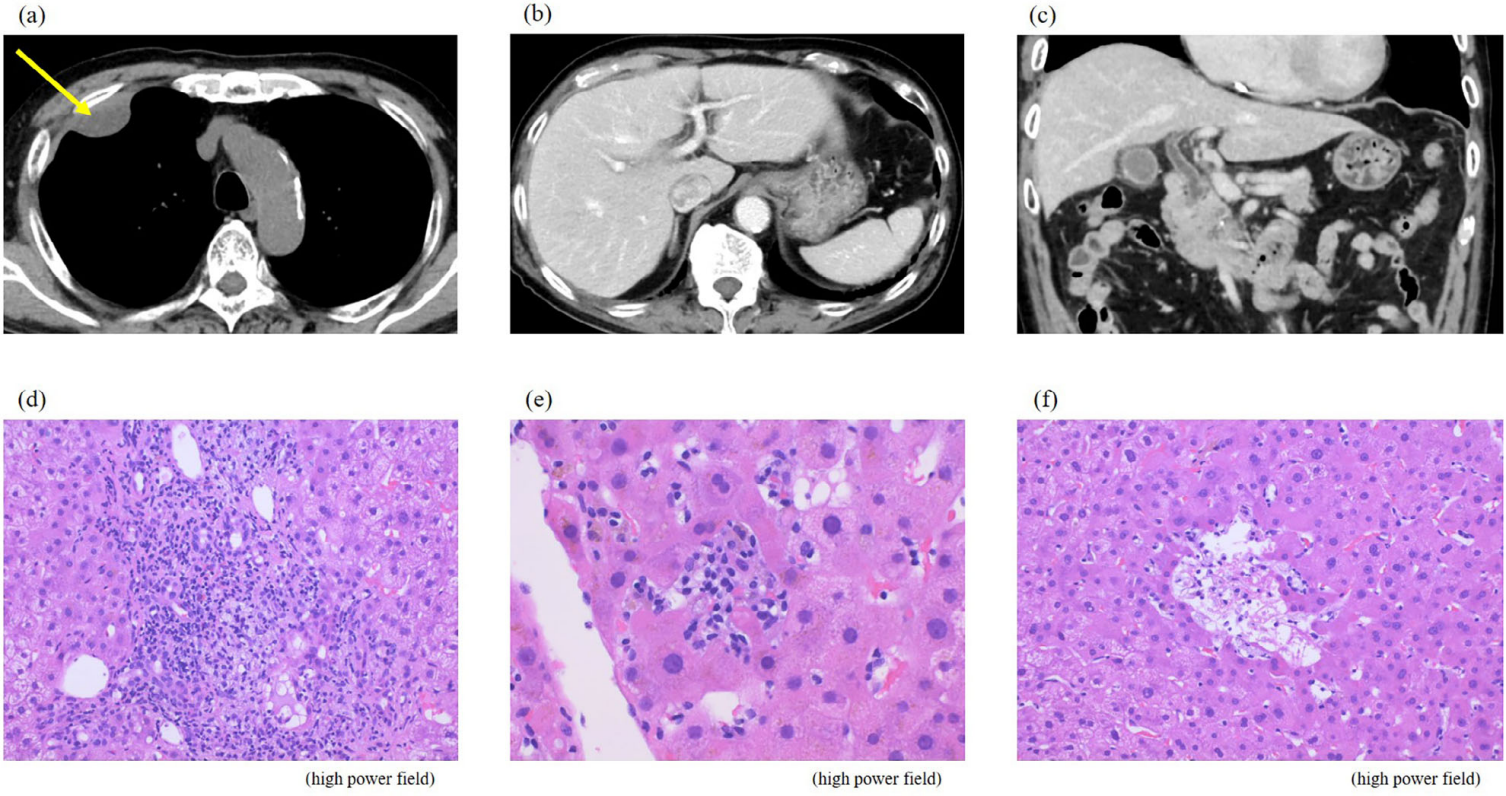
(a) Primary lesion of malignant pleural mesothelioma. (b) No hepatic abscess was found in the liver. (c) No common bile duct stones were found. (d) Liver biopsy showing interface hepatitis, (e) lipogranuloma, and (f) hepatocyte loss in the lobules; this finding was consistent with irAE hepatitis.

An 82‐year‐old man was treated with ipilimumab and nivolumab for malignant pleural mesothelioma (cT1N1M0, cStage II). On the 15th day after treatment initiation, significant liver dysfunction (aspartate aminotransferase [AST] 529 U/L and alanine aminotransferase [ALT] 499 U/L) was observed, and the patient was admitted to a hospital because of CTCAE grade 4 liver dysfunction. Treatment with prednisolone 60 mg/day (1 mg/kg/day) for irAE hepatitis was initiated, but after 1 week liver function did not improve (AST 434 U/L and ALT 727 U/L), and jaundice developed to CTCAE grade 3 (total bilirubin [T‐bil] 5.0 mg/dl). After starting steroid pulse therapy (3‐day administration of 1000 mg of methylprednisolone), the patient was transferred to our hospital for treatment. Blood tests showed elevated transaminase and bilirubin levels (CTCAE grade 3). Autoantibodies suggestive of autoimmune hepatitis and tests for viral hepatitis were negative (Table 1). Contrast‐enhanced CT showed no change in the malignant pleural mesothelioma lesion, and no findings suggestive of liver abscess or common bile duct stones were observed. However, his liver biopsy revealed a neuroinflammatory reaction, lipogranuloma, hepatocyte loss in the lobules, mild lymphocyte and neutrophil infiltration, interface hepatitis, and cholangitis in the portal region, which was consistent with irAE hepatitis (Figure 1). The patient was therefore diagnosed with irAE hepatitis. Remarkably, after steroid pulse therapy, bilirubin and transaminase levels decreased (AST 116 U/L, ALT 500 U/L, and T‐bil 2.0 mg/dl), and when the dose of prednisolone was reduced to 60 mg (1 mg/kg/day), jaundice did not worsen. However, transaminase levels increased again (AST 293 U/L and ALT 1115 U/L), therefore concomitant use of mycophenolate mofetil 2000 mg/day was started, and steroid pulse therapy was repeated because liver function did not improve. After repeated steroid pulse therapy, liver function improved (AST 68 U/L and ALT 441 U/L) and the dose of prednisolone was reduced to 120 mg (2 mg/kg/day). As with the previous time, there was concern that liver function would deteriorate again when prednisolone was reduced to ≤60 mg, so concomitant use of azathioprine 100 mg/day was started. Subsequently, hepatic function improved. Considering that the imidazole ring of azathioprine causes an allergic mechanism or dose‐dependent liver injury, we switched to mercaptopurine (30 mg/day). Since then, hepatic function has continued to improve (AST 26 U/L and ALT 44 U/L) and prednisolone was tapered to 10 mg/day (Figure 2).

**TABLE 1:** Blood test findings on hospital admission

Blood items	Value and Unit
White blood cell	10 440/μl
Red blood cell	435×10^4^/μl
Hemoglobin	12.5 g/dl
Platelet	18.4×10^4^/μl
C‐reactive protein	0.29 mg/dl
Total protein	6.9 g/dl
Albumin	3.6 g/dl
**Total bilirubin**	**5.9 mg/dl**
**Aspartate aminotransferase**	**363 U/L**
**Alanine aminotransferase**	**801 U/L**
Creatinine kinase	0.73 U/L
Sodium	140 mEq/L
Potassium	3.3 mEq/L
Chlorine	103 mEq/L
Blood urea nitrogen	15.8 mg/dl
Creatinine	0.73 mg/dl
C reactive protein	0.29 mg/dl
Prothrombin time	11.2 s
Activated partial thromboplastin time	28.7 s
D‐dimer	1.1 μg/ml
IgM‐hepatitis A virus antibody	(−)
Hepatitis B surface antigen	(−)
Hepatitis C virus antibody	(−)
IgA‐hepatitis E virus antibody	(−)
Antinuclear antibody	40 Titer

*Note*: Blood tests showed jaundice and liver dysfunction, but the results were not suggestive of viral hepatitis. Antinuclear antibodies were also negative. This is the liver function bold value that should be noted in this blood test data.

## DISCUSSION

Herein, we report a case in which mycophenolate mofetil was ineffective against steroid‐refractory irAE hepatitis and thiopurine‐based immunosuppressants were co‐administered to improve the patient's condition. It has been reported that the combined use of azathioprine and steroids was effective, but there are no reported cases of the combined use of mercaptopurine and steroids.^2^ Even though the guidelines published by the American Society of Clinical Oncology (ASCO) recommend corticosteroids as the first‐line treatment for hepatitis as an irAE of grade 2 or higher, they also recommend additional treatment with mycophenolate mofetil or azathioprine in cases where corticosteroids are not effective.^3^ In addition to the above, some studies have reported the use of infliximab for steroid‐refractory irAE.^4^ Azathioprine is known to be a prodrug of mercaptopurine, and both are used to treat autoimmune diseases, such as autoimmune hepatitis.^5^

Our patient was diagnosed with irAE hepatitis caused by ipilimumab and nivolumab. Prednisolone (1 mg/kg/day) did not suppress the deterioration of liver function, but steroid pulse suppressed it. Concomitant use of mycophenolate mofetil was initiated, but the worsening of liver function could not be suppressed. Eventually, the concomitant use of a thiopurine‐based immunosuppressive agent enabled the tapering of prednisolone, suggesting its effectiveness against irAE hepatitis. Azathioprine was also able to suppress the deterioration of liver function, but considering drug‐induced liver dysfunction due to azathioprine, it was switched to mercaptopurine. Mercaptopurine is a prodrug of azathioprine and was used because it was expected to have the same effect. In addition, mercaptopurine has shown efficacy as an immunosuppressive agent in inflammatory bowel disease.^6^ After switching to mercaptopurine, there was no worsening of liver function and the dose of prednisolone was reduced to 10 mg, which seems to have been effective. There is also a report that infliximab was effective against irAE hepatitis, and it was necessary to consider administering infliximab when thiopurine‐based immunosuppressants were ineffective.^7^ We therefore hypothesized that mycophenolate mofetil inhibits the purine biosynthetic pathway and exhibits immunosuppressive effects, and thiopurine‐based immunosuppressants exhibit immunosuppressive effects by suppressing purine and DNA synthesis. Although the exact mechanism is unknown, it is thought that thiopurine‐based immunosuppressants were effective in suppressing irAE hepatitis in this case due to the difference in the mechanism. In addition, we considered that prednisolone was gradually tapered smoothly due to the difference in the above‐mentioned mechanism and the increased dose of steroids. To our knowledge, this is the first report of improvement of irAE hepatitis by the concomitant use of a thiopurine‐based immunosuppressant, namely mercaptopurine, with prednisolone.

## AUTHOR CONTRIBUTIONS

Keita Kawakado: Conceptualization, data curation, writing – review and editing. Kazuhisa Nakashima, Tobita Hiroshi, Nagase Mamiko, Yukari Tsubata, and Takeshi Isobe: Writing – review and editing, supervision. Ken Yoshihara, Mika Horie, Megumi Hamaguchi, and Tamio Okimoto: Writing – review and editing.

## CONFLICT OF INTEREST STATEMENT

The authors declare no conflicts of interest.

## PATIENT CONSENT STATEMENT

Written patient approval was obtained.

## Data Availability

Data sharing is not applicable to this article as no datasets were generated or analyzed during the current study.
